# What constitutes a fulfilled life? A mixed methods study on lay perspectives across the lifespan

**DOI:** 10.3389/fpsyg.2022.982782

**Published:** 2022-09-30

**Authors:** Doris Baumann, Willibald Ruch

**Affiliations:** Department of Psychology, University of Zurich, Zurich, Switzerland

**Keywords:** fulfilled life, fulfillment, a good life, lay perspectives, lifespan, positive aging, mixed methods approach, positive psychology

## Abstract

Recently, we initiated a new research line on fulfillment in life by developing a conceptual framework and a self-report measure. To enhance conceptual clarity and complement theoretical considerations and empirical findings, we investigated lay conceptions of a fulfilled life in German-speaking participants at different life stages. First, we selected a qualitative approach using an open-ended question asking participants to describe a fulfilled life. Second, for a more comprehensive understanding, quantitative data were collected about the relevance of sources in providing fulfillment and ratings on a fulfilling life in the present and a fulfilled life in retrospect. Qualitative content analysis was used to assess the responses to the open-ended question. One-fifth of the data was double coded, and intercoder reliability was high (*Kappa* = 0.89). Responses comprised a variety of descriptions, and we grouped them into seven themes, three of which overlapped with the prior conceptualization, namely the core elements of (a) general description, (b) cognitive appraisals, and (c) affective appraisals. Cognitive evaluations related to intrapersonal aspects, particularly having lived life fully, attained personally significant goals, and developed oneself, as well as interpersonal, generative aspects, such as having made a contribution and been able to leave something of value. These categories are mostly in line with what the Fulfilled Life Scale (FLS) measures. Further themes referred to correlates—including (d) sources—and antecedents, including (e) resources, (f) personal characteristics, and (g) quality of life, all considered worthy to be the subject of empirical investigation but which were not included in the conceptual model. Qualitative and quantitative results suggest that individuals derive fulfillment from different sources. Fulfillment from a profession and having a life task was strongly associated with longer-term fulfillment. Only a few age and sex differences were found. The overarching framework developed from the qualitative results can stimulate further investigation. Our findings underscore that a fulfilled life as a distinct construct should be measured directly rather than via a proxy. Insights into the constituents, sources, and antecedents of a fulfilled life can inform practice to promote a life well lived.

## Introduction

Understanding what entails a fulfilling life and how to enhance it can be seen as a core mission in positive psychological research (Seligman and Csikszentmihalyi, 2000; Seligman, 2002). Until recently, however, a conceptual definition and a systematic investigation of the construct of fulfillment in life have been absent. To close this gap and initiate research, we introduced a theoretical framework of fulfillment in life (Baumann and Ruch, 2021) and an instrument to measure a fulfilled life in retrospect (Baumann and Ruch, 2022). Yet, the question of the extent to which individuals share the proposed scientific concept remains unanswered. Specifically, how do people themselves define a fulfilled life? What does a fulfilled life contain in lay people’s views? Indeed, it was argued that studying a phenomenon only from the outside is insufficient and that the lay perspective must also be considered (Furnham, 1988; Park and Peterson, 2007). Particularly in the early stages of a new research line, this information appears to be critical to understanding a concept and complementing theoretical perspectives (Delle Fave et al., 2011). Since research on fulfillment is only just beginning, it is crucial to establish what the term means to laypersons.

Prior to our work in clarifying the concept and meaning of fulfillment (Baumann and Ruch, 2021), the term was used merely as a synonym for well-being (e.g., Seligman, 2011) or as an umbrella term for positive outcomes that constitute the good life (e.g., Lopez and Snyder, 2003; Peterson and Seligman, 2004). Concepts of a good life and well-being have been operationalized in various ways (for an overview, see Huta and Waterman, 2014), such as life satisfaction (Diener et al., 1985), psychological well-being (e.g., Ryff, 1989), orientations to well-being (Peterson et al., 2005; Seligman, 2011), eudaimonic well-being (Waterman et al., 2010), or flourishing (e.g., Keyes, 2005; Schotanus-Dijkstra et al., 2016). Ryan and Deci (2000, 2001) provided another perspective with their self-determination theory (SDT) by describing the conditions that promote intrinsic motivation and well-being. To maintain self-actualization and healthy functioning throughout the lifespan, the three psychological nees for competency, autonomy, and relatedness must be satisfied. Although several approaches to the good life have emerged, a scientific approach to fulfillment in life has been lacking until recently. In this study, we aim to address the gap in the literature regarding lay conceptions of a fulfilled life. In this article, we first briefly introduce the multidimensional conceptualization of fulfillment in life and the recently developed and validated Fulfilled Life Scale (FLS; Baumann and Ruch, 2022). Second, to help locate the construct within a broader conceptual framework, we offer a brief review of findings from studies on lay conceptualizations, in which participants mentioned fulfillment as part of well-being concepts. In sum, although studies of lay conceptions have not yet examined fulfillment in life, there are few in which the term appears in the context of happiness and well-being.

### Conceptualization and assessment of fulfillment in life

In our theoretical conceptualization, we defined fulfillment in life as “a cognitive-affective experience referring to a sense of wholeness, fit, and value toward the self, one’s life, and one’s impact” (Baumann and Ruch, 2021, p. 6). The subjective experience consists of both cognitive-evaluative appraisals and affect. The cognitive component is represented in the Fulfillment in Life model (FiL), which is construed as a 3 × 3 matrix that includes criteria for fulfillment (wholeness, congruence, worthwhileness) and sources of fulfillment (self, life, impact/legacy), yielding nine cognitive facets. In terms of the whole lived life, “a fulfilled life refers to the positive appraisal of the person one has become, how one has led one’s life, and the impact one has made” (Baumann and Ruch, 2022, p. 2). The three criteria related to the self refer to the fulfillment derived from having made something of one’s potentialities, having been true to oneself, and having used one’s capabilities meaningfully. Combining the criteria with the source life describes fulfillment that comes from having lived fully and led a life that deeply suited one and has been worthwhile. The three criteria related to impact and legacy mean that one could make a positive difference in others’ lives, that one made contributions that reflected one’s values, and that one felt that one’s life mattered to others. The affective experience consists of positive low-arousal feelings, such as harmony, inner peace, deep inner contentment, or great gratitude (Baumann and Ruch, 2021). In addition, a fulfilled life is primarily free of deep regret, disappointment, and feelings of emptiness.

We proposed fulfillment in life as the generic term and distinguished between different time frames, ranging from a short-term to a long-term perspective. The four time frames differentiate between fulfillment in an activity, fulfillment in a role or life area, a fulfilling life encompassing the whole life at the present life stage, and a fulfilled life that, in retrospect, encompasses the whole lived life.

The outlined conceptualization served as the basis for developing the multidimensional FLS (Baumann and Ruch, 2022), which assesses the whole life in retrospect. Based on the data of two samples of 688 adults aged 40–93, we found that the nine cognitive facets can be measured independently and were highly reliable. Their intercorrelation indicated a structure with fewer factors and permitted the creation of an economic version. Through factor analyses, three factors were identified that were clear and easily interpretable. The final scale contains 24 items for the three cognitive dimensions and eight items for the affective experience of fulfillment:

*Unfolded Self and Life* items assess the perception that one has been able to develop, grow, be authentic, and live a full and true life (e.g., “I have had the courage to be as I really am.”)
*Positive Impact and Legacy* items measure the extent to which one has been able to positively impact the lives of others and live a life that mattered to the well-being of others (e.g., “I could make a positive contribution to other people’s welfare.”)
*The Worthwhile Life* items assess the perception that one’s pursuits have been worthwhile and that one has lived life well (e.g., “I have the certainty that I have lived for the right things.”)
*Fulfilled Life Affective Experience* items assess the presence of positive feelings related to the lived life (e.g., “I feel deep inner contentment.”) and the absence of negative emotions (“I feel rather empty.”—reverse scored).

Findings revealed that cognitive and affective fulfillment were needed to significantly increase the ability to predict a global fulfillment rating, which could not be sufficiently predicted by existing well-being measures. The scales were associated with several sociodemographic and contextual variables, including age, education, financial status, marital status, spirituality, self-rated health, or a good childhood experience.

### Fulfillment within lay conceptions of well-being

Prior research revealed that laypersons mention the term fulfillment in the context of conceptions of happiness and well-being. To our knowledge, fulfillment appeared in three studies that used an open-ended question or were in the form of sentence completion questions, and involved mature-aged persons (Ryff, 1989; Westerhof et al., 2001; Delle Fave et al., 2011). In the first study with a cross-national sample, fulfillment was cited in psychological definitions alongside other constructs such as harmony, emotions, well-being, achievement, or satisfaction (Delle Fave et al., 2011); it was not used synonymously but considered to be unique. The second study demonstrated that laypersons use fulfillment for global life evaluations that include meanings not covered by life satisfaction (Westerhof et al., 2001). In both studies, half of the statements referred to specific and contextual dimensions (e.g., interpersonal relationships, health, standard of living, or spirituality). Only in the third study was fulfillment described in more detail. Ryff (1989) examined conceptions of well-being among middle-aged and older individuals, with two questions related to what personal fulfillment and being unfilled mean. With respect to personal fulfillment, respondents in both age groups most frequently cited a sense of accomplishment and second most frequently stated enjoying life. Other themes included contributing, being a caring person, and having a good family life. Regrets were the most frequently mentioned theme regarding lack of fulfillment in both age groups. Besides, participants equated lack of fulfillment with unhappiness, loneliness, an empty life, no career, and not realizing talent. No significant age or sex differences were found in responses to these two questions. Although the concept of personal fulfillment is narrower than that of fulfillment in life, most of the components mentioned by participants are covered in the proposed conceptualization of Baumann and Ruch (2021).

In summary, when asked to define happiness or well-being, laypersons mentioned fulfillment as a separate concept and considered fulfillment for evaluating their lives as a whole. However, what laypersons mean by a fulfilled life remains to be clarified. Therefore, our aim is to study people’s ideas of a fulfilled life to advance conceptual clarity. To this purpose, we will use an open-ended question that allows respondents to express in their own words what a fulfilled life means to them. Participants will be asked to give a description of their most telling example of a fulfilled life, regardless of what their life is like. To gain different types of information about the conceptions and experiences of a fulfilled life, we will employ a convergent mixed methods design. This ensures greater depth and diversity of responses than a purely quantitative approach. Furthermore, the mixed methods approach permits us to obtain and compare different perspectives from qualitative and quantitative data and gain a more comprehensive knowledge of the construct than would be possible with an isolated method (Creswell, 2014). Findings can help expand the understanding of the nature of a fulfilled life, strengthen theory-building and empirical research, and be relevant to practice. For individuals, clues to the characteristics of a fulfilled life can help set the right course at a younger age or initiate midlife corrections to focus on what is deemed valuable and successful in a final life review. In addition, a knowledge of sources of fulfillment can guide people in creating a more fulfilling life by engaging in and cultivating specific activities and roles. Furthermore, professionals (e.g., coaches, counselors) working with individuals in a particular life stage can benefit from a nuanced knowledge of their target group’s subjective perspective.

### The present study

The main objective was to examine lay perspectives of a fulfilled life to better understand this previously under-researched construct. The objectives of the qualitative approach were to (1) investigate the content of lay perspectives of a fulfilled life across the lifespan, (2) examine age group and sex differences, and (3) assess the consistency of the mentioned content with the scientific conceptualization and the FLS proposed by Baumann and Ruch (2021, 2022). Both the scientific conceptualization and the instrument should sufficiently converge with lay understanding. As part of the quantitative approach, we used ratings to explore the relevance of various sources (e.g., profession, life task, spirituality) in providing fulfillment and how these relate to ratings of a fulfilling life in the present and a fulfilled life in retrospect. We assumed that fulfillment from roles in life and activities (sources) have a stronger impact on a fulfilling life at the current life stage than on a fulfilled life in retrospect. Therefore, we expected stronger relationships between the sources and the ratings of a fulfilling life in the present than a fulfilled life in retrospect. We relate and compare the findings from both data sources.

## Materials and methods

### Participants

Sociodemographic characteristics of the German-speaking participants (total sample: *N* = 747, subsample: *N* = 126) are presented in Table 1. As part of a larger research project on fulfillment in life, we aimed for a broad sample spread by age and gender, from which we could then draw a subsample for the qualitative study. The participants were randomly selected to ideally include 20 persons per age decade and to attain a balance of sex. The age group 80 years and older included only six participants.

**Table 1:** Sociodemographic characteristics of participants.

Characteristics	Subsample	Full sample
Age (years)		
Range	20–93	18–93
Mean (SD)	50.48 (19.12)	49.84 (16.48)
Sex		
Female	66 (52.4%)	594 (79.5%)^a^
Highest educational level		
Compulsory school qualification	–	9 (1.2%)
Vocational education/training	14 (11.1%)	92 (12.3%)
General education (baccalaureate)	24 (19.0%)	141 (18.9%)
Diploma of professional education	17 (13.5%)	138 (18.5%)
University or university of applied science	71 (56.4%)	367 (49.1%)
Marital status		
Single, never married	57 (45.2%)	271 (36.3%)
Married or registered partnership	50 (39.7%)	306 (41.0%)
Separated	2 (1.6%)	25 (3.3%)
Divorced	11 (8.7%)	118 (15.8%)
Widowed	6 (4.8%)	27 (3.6%)
Being a parent	65 (51.6%)	412 (55.2%)

*N*_Subsample_ = 126. *N*_Full Sample_ = 747.

a Three participants did not indicate their sex.

### Data collection

Qualitative and quantitative data collection was conducted concurrently and in German via an online survey. We recruited participants through voluntary organizations and online advertisements. They were not paid but could download a leaflet with suggestions for promoting their mental health. Informed consent was obtained from all study participants.

### Qualitative instrument

#### Open-ended question

Lay perceptions of a fulfilled life were assessed with the open-ended question (which comprised several prompts): “What makes for a fulfilled life? When you look back on life, you may find it to be variously empty or full. What does a very fulfilled life look like? Please describe below the example of a fulfilled life that is most true for you. This can be an example from someone you have read about or heard about. Your example can also be about someone you know personally. It can also be an example that you merely imagine/that comes to mind. It can be based on your own life that you have lived as it has actually been and still improved by the things you wish would be there. All in all, the idea of a fulfilled life as you wish it for yourself and your loved ones. (Describe in about 5–10 sentences).”

#### Quantitative instruments

The ratings of a Fulfilling Life in the present and a Fulfilled Life in retrospect are single-item measures developed for the research project on fulfillment in life. These ratings were anchored in the brief description participants gave in their responses to the open-ended question in this study and in an example, they gave of a fulfilling life in the present (not included in this study). Concerning their examples, respondents rated the two statements on an 11-point rating scale (0 = *not at all fulfilling/fulfilled* to 10 = *entirely fulfilling/fulfilled*). Example item: “Compared to your given example, how fulfilled is your life lived so far in retrospect?”

To investigate sources of fulfillment, participants rated 16 roles and activities according to their relevance in providing fulfillment on a 7-point rating scale (1 = *not fulfilling* to 7 = *very fulfilling*). The list included the following 16 sources: profession, volunteer work, partnership, parenting, family, grandparenting, spirituality/religion, hobby, learning and personal development, challenges, life task, reaching a life goal, friendship and social network, creative expression, traveling, and nature. Participants could also select the category of “not applicable.” The roles and activities were identified through a previous literature review (Baumann and Ruch, 2021), which gave indications on sources of fulfillment.

Sociodemographic questions were asked about age, sex, education, marital status, being a parent, and being a grandparent.

### Coding procedure

**Figure 1:**
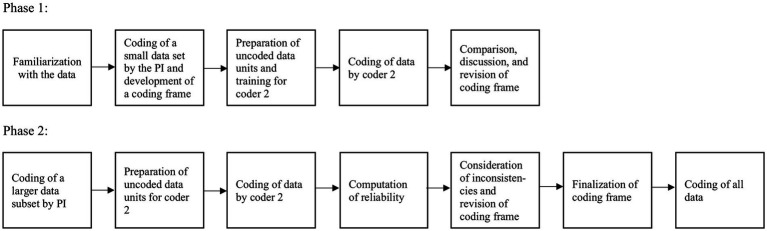
Flowchart of the various steps taken to analyze the data from the open-ended question. PI, Principal Investigator.

We followed the data analysis strategy outlined by O’Connor and Joffe (2020) and depicted in Figure 1. Before coding the data, the following preliminary decisions were made: (1) a principal coder and a second coder would analyze the data using qualitative content analysis, (2) 20% of the data is double coded, (3) the unit of code comprises conceptually meaningful parts, (4) depth of coding contains manifest meaning, (5) codes are generated inductively, (6) only one code is assigned to multiple mentions of the same aspect from a participant, and (7) threshold of intercoder reliability is assessed as a Brennan and Prediger’s Kappa (1981), from 0.61 as substantial and from.81 as very good (Landis and Koch, 1977). During the coding process, the coders were not informed about the personal characteristics behind the data. Qualitative analysis was performed with MAXQDA software. After familiarizing herself with the data, the principal investigator assigned codes to a part of the data through an inductive process and developed a hierarchical coding frame. Code memos were used to define the meaning and content of a code and to differentiate it from others. The codes were sorted into subcategories and categories, and themes were generated for the underlying meaning of the categories. It became apparent that participants mentioned not only elements of a fulfilled life, but also prerequisites and sources. Therefore, statements were examined according to this differentiation. For instance, when an individual mentioned the topic of partnership, we inspected whether the person meant the fulfillment drawn from this role (source of fulfillment) or the aspect of a fulfilled life in having attained a vital life goal (e.g., having found a partner, having a long-lasting relationship). Regarding contentment, we tried to distinguish whether participants meant the feeling or the evaluation. Satisfaction with one’s professional achievements was treated as an evaluation, while becoming a contented person or inner contentment was considered as affect.

The principal investigator trained the second coder and explained the coding frame. Then the second coder analyzed the set with the uncoded *a priori* defined data units. Subcategories, categories, and themes were examined and were evaluated regarding consistency. The principal investigator and the second coder reviewed and discussed deviations until a consensus was established to ensure the discriminatory power of the categories and to improve the coding frame. Afterward, the principal investigator coded a larger data subset. The second coder again received a data set with uncoded data to apply the codes. To assess intercoder reliability, we calculated a chance-corrected coefficient, which was high, *Kappa* = 0.89. For any discrepancies found, the cases were discussed until an agreement was achieved. The principal investigator coded the remaining data.

## Results

### Qualitative analyses

#### Overarching framework of a fulfilled life

**Figure 2:**
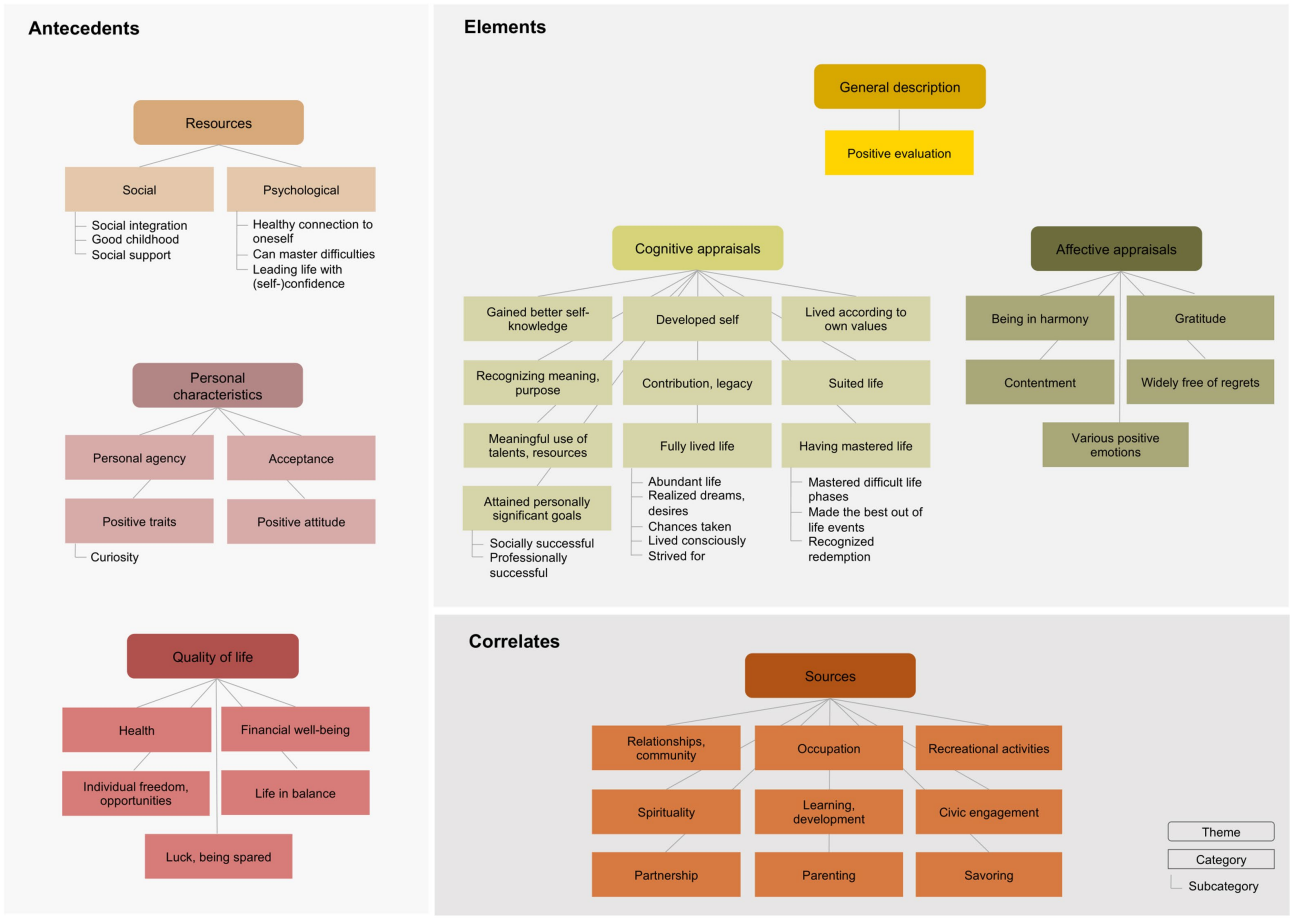
Overarching model of a fulfilled life from lay perspectives.

We labeled 504 meaning units with a code, collated them into subcategories, categories, and themes, and then presented them in an overarching framework (see Figure 2). We distinguished between essential core elements of a fulfilled life (essential to the meaning of a fulfilled life) and aspects classified as correlates and antecedents. They are represented in three separate blocks in the figure. Core elements comprised a general description at the most abstract level and included cognitive and affective appraisals of a fulfilled life. Correlates entailed various sources from which participants derived fulfillment and contextual aspects that could be considered antecedents, including quality of life, resources, and personal characteristics. The content of each theme, category, and subcategory as well as translated example quotes are presented in Table A of the Supplementary material of this article. Table A also displays the frequencies of the statements. About half of the statements referred to core elements of fulfillment, including a general description, cognitive, and affective appraisals. At the level of themes, most statements were made about cognitive appraisals (39.5%), followed by sources (19.3%), affective appraisals (10.3%), quality of life (9.5%), personal characteristics (8.7%), resources (8.3%), and general descriptions (4.4%). The two categories, a fully lived life and having attained personally significant goals, received the most statements by both men and women, by approximately one-third of participants each.

#### Elements of a fulfilled life

The first block in Figure 2 comprises the elements we identified as essential to the meaning and definition of a fulfilled life. Core were the three themes of general description, cognitive appraisals, and affective appraisals.

#### General description

This theme refers to participants’ descriptions of a fulfilled life at the most abstract level. Statements included that a fulfilled life could look different for everyone and that a life considered fulfilled from an outside view can feel like a failure to a person or vice versa. Respondents also mentioned that old age means fulfillment and that reaching an older age makes it possible to assess whether one’s life is fulfilled. A fulfilled life also includes setbacks, because the negative feelings allow for comparison, so mistakes would also belong to a fulfilled life:

“If I can say on my deathbed that I’ve had a fulfilling life, that would certainly be nice. That doesn’t have to be a life of prosperity or publicity or.., I have to feel the life I lived was fulfilling. Was beautiful the life with all the ups and downs.” (Female, 72)^1^

Within this theme, the category of *positive evaluation* referred to statements that emphasized that positive things are in the foreground and predominate. This includes the idea that one has done more right than wrong with regard to one’s decisions and behavior, and that the path one has chosen in life has brought more joy than sorrow:

“When I imagine that I am old and look back on my past life, I would like to come to the realization that despite all the mistakes I have made during my life, overall, I am satisfied with the way I have spent it.” (Male, 24)

#### Cognitive appraisals

This theme comprised 10 categories of evaluative statements. The largest of these related to a *fully lived life,* which entailed five subcategories. The first, *abundant life,* emphasized the richness and wealth of experiences that are part of a fulfilled life. One experienced a lot, had exciting encounters with people, was able to discover the world, and retains many fond memories:

“A fulfilled life is when you have seen and experienced many things.” (Female, 21)

Other subcategories contained the aspects of *having realized dreams and desires, taken chances, lived consciously,* and *strived for. Attained personally significant goals* was the second-largest category:

“A fulfilled life allows one to look back in old age on a large number of personal successes and achievements that were important to oneself. These are not necessarily financial successes, but rather inconspicuous things that perhaps only in retrospect turned out to be successes. Or paths despised by others, which one wanted to follow at all costs, which did not bring one to the planned goal, but perhaps to an unplanned, much greater goal.” (Female, 52)

This category comprised two subcategories divided into *socially* and *professionally successful*. Social success related to having been able to find a partner, start a family, have a happy family, build a solid and long-lasting circle of friends and good relationships, and see the positive development of the offspring:

“So, for example, three healthy, intelligent and satisfied children, whom you have raised to become capable human beings and valuable members of society, is a symbol of a fulfilled life for me as a mother. If one has managed to maintain one’s partnership through ups and downs and to one’s own satisfaction, if one has struggled and suffered and yet is still together, then that is a symbol of fulfilled life for me.” (Female, 54)

Professional success was mentioned in general terms and was also described as satisfaction with what has been achieved professionally and having attained in one’s career what one wanted. *Developed self* was another major category within the cognitive appraisals referring to how a person could learn, develop, and grow. It describes a person’s ability to make progress in various areas, learn from mistakes, experiment, develop and use strengths, and redefine oneself:

“I have become closer and closer to the person I want to be.” (Female, 30)

Participants considered a *contribution and legacy* as part of a fulfilled life. This category suggests that a positive impact on the lives of others can take many forms. For example, respondents’ statements related to having raised children, having left an asset that generates profit across generations, and being able to look back on grandchildren and great-grandchildren. Having passed on learnings from one’s life and what is essential for a good life were also mentioned. Other descriptions included having done something meaningful and valuable for society and the environment, helped people, given back, and supported persons vital to oneself:

“One has left traces. One has moved the world, even if only in small pieces.” (Male, 54)

The category of *having mastered life* was composed of three subcategories. The first was *having mastered difficult life phases*, including having coped with crises and overcome low points in life:

“A fulfilling life includes difficulties that had to be overcome. Without the endangerment of the circumstances recognized as valuable, I as a human being could not feel the emptiness as a counter design, from which a fulfilling life stands out.” (Male, 34)

The second subcategory emphasized having *made the best out of life events,* situations, and setbacks and the knowledge of this. The third subcategory referred to *recognized redemption,* in perceiving that adversity turned into something good, a painful experience produced a strong sense of justice, or something that one would have chosen differently turned out well. Another category pertained to *recognizing meaning and purpose.* It described the perception that one has fulfilled one’s purpose in life, had a life task, and—in retrospect—recognized meaning in everything, including one’s actions:

“So, the certainty of having led a meaningful life.” (Male, 44)

The category referring to *a suited life* entailed the feeling of having been true to oneself, having made good decisions, having taken the right path in life, and having pursued one’s own ideas and interests, as well as having found ways to combine and apply them with their talents. Within the category having *lived according to own values,* statements described specific values that one would like to see realized. These include that one could be there for others, having been a role model for others, having acted morally right for oneself, or having lived honestly. The other two categories within cognitive appraisals were *gained better self-knowledge,* i.e., seeing oneself and one’s inclinations, weaknesses, and strengths more clearly, and *meaningful use of talents and resources,* which describes a fulfilled life in which one did not waste time and energy, but feels one has used them properly.

#### Affective appraisals

In addition to the cognitive appraisals, participants provided affective descriptions of a fulfilled life, which we grouped into five categories. The largest category within the theme related to *being in harmony.* It was about being at peace and in harmony with oneself, one’s life circumstances, or the environment:

“A fulfilled life is when you are reconciled with your life in old age.” (Female, 84)

In addition, the feeling of *gratitude* was part of participants’ descriptions, which included statements such as looking back on one’s life with great gratitude, feeling deep gratitude, and thankfulness for what is and for what one has received:

“A full life contains much gratitude and appreciation.” (Female, 32)

Another category concerned *contentment,* which entailed great inner contentment, contentment with oneself, one’s life, what one has experienced, and having become a contented person. The category *widely free of regret* described a fulfilled life, looking back without regretting not having done something or regretting important decisions and experiences, and without having to regret anything (in retrospect):

“For me, a fulfilled life is when I could die without regrets, with a great inner satisfaction.” (Female, 30)

Included in the category of *various positive emotions* are feelings of pride, inner peace and serenity, and a joyful looking back on the experience.

### Correlates of a fulfilled life

#### Sources

Participants’ statements referred to sources from which they draw fulfillment, which we grouped into nine categories, displayed in a separate block in Figure 2. The principal source was *relationships and community,* with the aspect of sharing time and life. Above all, the quality and depth of a relationship seems to enrich and fulfill life. *Occupation* was another main source from which people gain fulfillment, especially if the occupation provides joy, makes one happy, and fits with one’s calling:

“I want professional fulfillment in a job where I add value to others through my skills.” (Female, 32)

The category *recreational activities* encompassed leisure pursuits, such as engaging in music, experiencing nature, sexual fulfillment, physical exercise, or enjoying small things. In addition, people find fulfillment in learning and development, such as learning new perspectives and meeting new people, being challenged by different tasks and responsibilities, and learning new things. Participants cited social roles as sources of fulfillment, such as a fulfilling *partnership* or *parenting*. Lived *spirituality* and *civic engagement* were two other categories we identified. Lastly, *savoring* was mentioned as a source of fulfillment, with participants describing enjoying beautiful and magical moments.

### Antecedents: contextual aspects

Participants’ descriptions also involved various aspects that facilitate a fulfilled life, including resources, personal characteristics, and quality of life (see Figure 2).

#### Resources

This theme included the two basic categories of *social* and *psychological resources,* which were seen as prerequisites for a fulfilled life. Three subcategories pertained to social resources. First, *social integration* means having loving people around, having a stable family and good friendships, enjoying a trusting network of relationships inside and outside the family, and having a sense of belonging. The second subcategory referred to a *good childhood.* It was described as carefree and sheltered, but not overprotected, in which one experienced love and support, and felt cared for. The third category on *social support* subsumed mentions such as support from different people, a family that provides a harbor in stormy times and confers stability, and friends one can rely on, whom one can count on and who stand by one’s side:

“… socially integrated are important prerequisites.” (Female, 71)

Psychological resources also consisted of three subcategories, the first being *a healthy connection to oneself.* Statements were made about connectedness with oneself, self-forgiveness, and self-acceptance:

“When you don’t let yourself be too unsettled by external factors in difficult times and have found a secure anchor within yourself.” (Female, 42)

A second subcategory labeled *can master difficulties* meant the ability to cope with difficulties and find solutions. The third subcategory concerned *leading life with (self-) confidence,* which included the idea that everything will occur as it must.

#### Personal characteristics

Participants named various personal qualities that facilitate a fulfilled life. The category of *positive traits* included gratitude for one’s life and what one has, modesty and realism, courage, and never giving up. Of note was *curiosity*, for which we formed a subcategory. Another group of statements was subsumed under the category of *personal agency* and referred to taking personal responsibility, developing creative initiative, being active, and shaping one’s life. Not holding anyone responsible for one’s own happiness and the ability to say no were other statements included in the responses:

“You can’t live a full life without doing it yourself.” (Female, 75)

*Acceptance* and *a positive attitude* were other characteristics that respondents addressed, and for each of them, we created a category:

“Also being able to accept things in life that you would do differently today.” (Female, 83).

“We see everything with somewhat positive attitudes.” (Male, 93).

#### Quality of life

Respondents referred to the overall quality of life, including subjective perceptions and excellent living conditions. Statements were also made about specific components, such as *health* and *financial well-being*—the latter referring to having enough money, no existential hardships, and financial security. Another category dealt with the issue of *individual freedom and opportunities,* including the ability to make independent decisions and choose one’s profession and place of residence, as well as the possibility of receiving a good education:

“I grew up in a time that offered me excellent opportunities, possibilities, and conditions for a fulfilling life.” (Male, 70)

Quality of life also involved the category of *life in balance.* Participants emphasized the aspects of having enough free time and achieving a balance between work and private life without burning out. The last category within this theme related to a minor but insightful topic, namely the fact of *luck and being spared.*

#### Lay perspectives across the lifespan and age group differences

Looking more closely at the age categories by decade, several results are worth highlighting. Participants in their 20s and 30s made most statements about the fully lived life: 13 and 14 participants, respectively, out of 20 participants per decade. The subcategory of abundant life was the largest in both age groups, mentioned by seven and six participants each. Having attained personally significant goals was mentioned most frequently among those aged 40–79, with nine participants aged 40–49, seven participants aged 50–59, eight participants each aged 60–69 and 70–79. Equally or second-most mentioned were: a fully lived life, in the 40–49 age group (seven participants), a contribution/legacy as well as being in harmony in the 50–59 age group (six participants each), social resources in the 60–69 age group (eight participants), and developed self in the 70–79 age group (six participants).

We used the coded data as categorical variables in SPSS to investigate group differences and examined the data when aggregated, as with themes and some categories. Because many different aspects were mentioned, we created a younger age group (20–49 years) and an older age group (50+). Forming groups for each decade would have resulted in too few participants for analyses. Two-tailed Fisher’s exact tests were used for categorical data and Mann–Whitney tests for aggregated data. There were a few age differences.^2^ The younger age group (20–49) mentioned the category of a fully lived life more often than the older age group (50+). A Mann–Whitney test indicated that this difference was significant, *U*(*N*_younger_ = 60, *N*_older_ = 66) = 1419.00, *z* = −3.45, *p* = 0.001, *r* = 0.31. Within this category, younger individuals more frequently addressed having had an abundant life (*p* = 0.013, two-tailed Fisher’s exact test, Cramer’s *V* = 0.24) and having taken chances (*p* = 0.027, two-tailed Fisher’s exact test, Cramer’s *V* = 0.21) than older adults. On the other side, the older age group emphasized positive traits as a condition for a fulfilled life more than young adults did *U*(*N*_younger_ = 60, *N*_older_ = 66) = 1687.50, *z* = −2.48, *p* = 0.013, *r* = 0.22.

#### Sex differences

There were only a few significant differences between men and women in their perceptions of a fulfilled life (see footnote 2). First, only men mentioned the aspect of luck and being spared (*p* = 0.049, two-tailed Fisher’s exact test, Cramer’s *V* = 0.19). Second, women stated having mastered life more frequently than men. A Mann–Whitney test indicated that this difference was significant, *U*(*N*_women_ = 66, *N*_men_ = 60) = 1630.00, *z* = −2.70, *p* = 0.007, *r* = 0.24. Within this category, women more often stated having made the best out of life events as an evaluation for the fulfilled life than men did (*p* = 0.029, two-tailed Fisher’s exact test, Cramer’s *V* = 0.21).

### Quantitative analyses

#### Rated sources of fulfillment

Using the quantitative approach, we aimed to examine participants’ subjective experience of the relevance of each role and activity for fulfillment. Ratings for volunteering, parenting, and grandparenting were only included if individuals actually performed these roles. Table 2 displays the mean levels of fulfillment resulting from roles and activities, in descending order. In general, all categories seem to represent relevant sources of fulfillment, as the values are above the scale midpoint. Spirituality yielded the lowest level of fulfillment and the highest standard deviation of all sources. However, a separate analysis revealed that the mean level of fulfillment for spirituality was 5.73 for individuals who considered spirituality important and 6.65 for persons considering spirituality very important.

**Table 2:** Descriptive statistics and correlations for fulfilling life and fulfilled life ratings and level of experienced fulfillment in activities and roles.

Ratings	*M*	*SD*	1	2
1. Fulfilling life (present)	6.98	2.01		
2. Fulfilled life (in retrospect)	7.13	1.93		
Fulfillment in roles, activities				
Nature	6.23	1.15	0.24***	0.18***
Grandparenting	6.20	1.24	0.27**	0.26*
Parenting	5.99	1.27	0.31***	0.28***
Learning, personal development	5.92	1.20	0.38***	0.32***
Volunteering	5.90	1.16	0.22***	0.21**
Hobby	5.80	1.26	0.36***	0.24***
Friendship, social network	5.63	1.35	0.39***	0.33***
Reaching a life goal	5.61	1.46	0.43***	0.35***
Partnership	5.51	1.75	0.45***	0.29***
Challenges	5.46	1.36	0.42***	0.32***
Life task	5.46	1.49	0.51***	0.45***
Family	5.44	1.56	0.34***	0.35***
Travelling	5.42	1.54	0.19**	0.19**
Creative expression	5.35	1.46	0.27***	0.24***
Profession	5.28	1.60	0.53***	0.42***
Spirituality, religion	4.30	1.98	0.24***	0.21***

*N* = 98–735 due to category of ‘not applicable’ and data selection of actually holding the role as a volunteer, parent, or grandparent.

*p < 0.05; **p < 0.01; ***p < 0.001.

#### Age group differences

To explore potential differences in the ratings, the size of the sample allowed us to divide the participants into three age groups (18–39, 40–64, 65+). Analysis of variance (ANOVA) indicated significant group differences in the categories of profession, *Welch’s F*(2, 181.01) = 13.36, *p* < 0.001, *η^2^* = 0.04, spirituality, *F*(2, 617) = 17.36, *p* < 0.001, *η^2^* = 0.05, learning and personal development *Welch’s F*(2, 341.11) = 6.49, *p* = 0.002, *η^2^* = 0.02, life task *F*(2, 662) = 6.78, *p* = 0.001, *η^2^* = 0.02, and nature *F*(2, 724) = 6.00, *p* = 0.003, *η^2^* = 0.02. To determine significant differences among the groups, we used Games-Howell and Hochberg GT2 post-hoc test. Regarding profession, age group 40–64 (*M* = 5.35, *SD* = 1.67) and age group 65+ (*M* = 5.94, *SD* = 1.51) experienced this source as providing significantly more fulfillment than the youngest age group 18–39 (*M* = 4.90, *SD* = 1.38). The oldest age group gained significantly more fulfillment from this source than did the middle group. Furthermore, the two older groups derived significantly more fulfillment than the youngest group regarding the following four sources, spirituality: age group 65+ (*M* = 4.80, *SD* = 1.80), age group 40–64 (*M* = 4.47, *SD* = 1.93), age group 18–39 (*M* = 3.55, *SD* = 2.03); learning and personal development: age group 65+ (*M* = 6.07, *SD* = 1.04), age group 40–64 (*M* = 6.01, *SD* = 1.19), age group 18–39 (*M* = 5.66, *SD* = 1.29); life task: age group 65+ (*M* = 5.74, *SD* = 1.42), age group 40–64 (*M* = 5.51, *SD* = 1.46), age group 18–39 (*M* = 5.14, *SD* = 1.54); and nature: age group 65+ (*M* = 6.39, *SD* = 0.94); and age group 40–64 (*M* = 6.29, *SD* = 1.15), age group 18–39 (*M* = 5.99, *SD* = 1.24).

#### Sex differences

To test whether men and women differed on the level of fulfillment regarding the categories, we conducted *t*-tests using bootstrapping procedures. Only for the category friendships and social network, was a significant difference found. Regarding this category, women reported higher means (*M* = 5.70, *SD* = 1.35) than men (*M* = 5.39, *SD* = 1.34). This difference, −0.31, BCa 95% CI [−0.540, −0.078], was significant *t*(730) = −2.48, *p* = 0.013, Cohen’s *d* = 0.23.

#### Relationship between sources, a fulfilling life, and a fulfilled life

First, we investigated the associations between the fulfilling life and fulfilled life ratings, age, and sex. We found a small positive correlation between age and a fulfilling life (*r* = 0.26, *p* < 0.001), and age and a fulfilled life (*r* = 0.26, *p* < 0.001). There was no significant relation between sex and a fulfilling life (*r* = −0.03, *p* = 0.49) or sex and a fulfilled life (*r* = −0.06, *p* = 0.14). We further calculated correlations between a self-rated fulfilling life at present, a fulfilled life in retrospect, and the rated categories for experiencing fulfillment. Strength of association ranged between small and medium (see Table 2). The numerically highest correlation coefficients were found for the categories of profession, life task, partnership, reaching a life goal, and challenges. As expected, greater correlations were observed for a fulfilling life at the present life stage than for a fulfilled life in retrospect.

## Discussion

This is the first study to examine lay conceptions of a fulfilled life. From participant responses, we gained insights into the central and essential elements of the construct, which contributed to greater conceptual clarity. Our findings confirmed the multifaceted nature of the concept, as participants mentioned both cognitive and affective aspects. Moreover, the results suggest that our proposed model and the factor structure discovered may reflect lay people’s understanding of a fulfilled life. We obtained additional evidence for the need to treat a fulfilled life as a distinct and measurable concept. Despite not being specifically questioned, participants made statements regarding correlates and antecedents. Thus, a comprehensive picture of the content and context of a fulfilled life emerged. The mixed methods approach allowed us to further explore the context, particularly the relevance of different sources and their relationship to longer-term fulfillment. In the following, we discuss the qualitative results on the core elements and draw a comparison with the proposed conceptual model and structure. We then discuss the sources and their relevance, and integrate findings from our qualitative and quantitative approaches. Finally, we place a fulfilled life in a broader context, as has emerged from the previously mentioned antecedents, and consider a fulfilled life from a lifespan perspective.

### Core elements of a fulfilled life from a lay perspective

Qualitative findings revealed that at the most abstract level, participants described the subjective nature and the challenging sides of life that make a fulfilled life conscious. From the lay perspective, a fulfilled life is not the perfectly lived or the carefree life, but positive aspects prevail within all the complexities of life. The way individuals interpret their life experiences, such as viewing low points in life as opportunities for growth, may play a role in this evaluation and help to foster maturity and well-being (Bauer et al., 2005). Moreover, a sense of meaning is achieved by creating an evolving and coherent life story; McAdams (2001) refers to this as narrative identity. Lay perspectives referred to both cognitive and affective appraisals, with more statements referring to the cognitive aspects. From this, we can conclude that the evaluation of whether life is fulfilled is based on different criteria. Hence, the cognitive core elements reveal what counts for people to arrive at this conclusion, and the affective elements indicate the feelings involved. Laypersons consider a fulfilled life primarily as a fully lived life, as evidenced by the frequency of statements. It describes the richness of a lived life, chances taken, or a life led consciously. Such a life points to the depth (richness) and the degree of realization and completeness. From the lay perspective, almost as relevant for a fulfilled life is that personally significant goals have been attained. As regards the specificity of goals, participants notably stated the attainment of social and professional goals. The emphasis lies on the meaningfulness and value of these goals. Life appears enriched, rewarding, and complete through having been able to build a family, maintain long-lasting relationships, or look back on a successful career. Another central facet of a fulfilled life was that one could develop and evolve as a person. In a previous study, failure to realize talent was equated with a lack of fulfillment (Ryff, 1989). Contributing and leaving something of value for others constituted an additional core feature. This finding demonstrated that a fulfilled life is not a self-centered life but emerges from being able to transcend self-interest and develop the virtue of care (Erikson, 1985; Ryff, 1989). The fulfillment that comes from promoting the welfare of others might be based on the values of universalism and benevolence, which belong to the value type of self-transcendence described in Schwartz’s (1994) value theory. In comparison to the life evaluation operationalized as life satisfaction, a fulfilled life includes the contentment of positively impacting upon others’ lives. Recognizing meaning in life and perceiving the resulting good were addressed as part of a fulfilled life. Furthermore, participants viewed a fulfilled life as a life that has suited and was lived according to one’s standards. A life judged as fulfilled also seems to depend on whether one could master difficult life phases and make the best of adverse events, which was mentioned more frequently by women than by men.

Laypersons referred to an affective side of a fulfilled life by describing different feelings. One of these was inner peace and harmony, an aspect mentioned in a previous study on people’s happiness (Delle Fave et al., 2011). Participants mentioned contentment in the context of affective experience on the one hand, but also for evaluation purposes. In coding, we attempted to differentiate accordingly. In addition, participants suggested that a fulfilled life went along with feelings of gratitude. It should largely exclude regret, equated with a lack of fulfillment in previous findings (Ryff, 1989).

### Convergence of lay perspectives and conceptual and operational definitions

The conceptual model covers most of the categories that emerged from participants’ statements regarding the substantive conceptual components. The core elements included evaluative (e.g., a life lived according to one’s values) and affective (e.g., being in harmony) content, as suggested by the conceptual and operational definitions. More statements were subsumed under cognitive appraisals than affective experience, indicating the different facets of evaluative fulfillment. These results demonstrate the complexity of the construct and that conceptualization and operationalization must represent the cognitive experience as multidimensional. The categories of cognitive appraisals can be summarized in terms of contentment with one’s own development (categories: gained better self-knowledge, developed self, meaningful use of talents and resources), with one’s lived life (categories: lived according to own values, suited life, fully lived life, attained personal meaningful goals, recognized meaning and purpose), and with one’s contribution to the welfare of others or a worthy cause (category: contribution/legacy). These aspects can be found in the postulated three sources (self, life, impact/legacy) of the FiL model. The proposed criteria for a fulfilled life (wholeness, congruence, meaningfulness) are reflected within different categories. For example, the criterion of wholeness and completeness is found in the category of a fully lived life, congruence is part of the category a suited life, and meaningfulness was featured in the categories recognizing meaning and meaningful use of talents and resources. It should be noted that the category of having mastered life is not explicitly represented in our model. However, this notion can be covered by the conceptualization that a fulfilled life consists of having become a whole and complete person and being able to look back on a life well lived and to recognize meaning in difficult periods of life. Nevertheless, these results show that the elements of a fulfilled life derived from lay perspectives essentially correspond with the proposed theoretical model and are substantially represented by the subscales of the FLS. Hence, future research can build on the proposed model and employ the FLS, as it allows the construct to be measured directly rather than via a proxy, as has been done in the past.

Given the number and variety of mentioned aspects, we can conclude that a model and a measure are best built at an abstract level, as we have suggested, because otherwise it would not be possible to cover all the facets of what individuals means by a fulfilled life. For instance, the subcategory socially successful is not explicitly part of the model. The goal of our model was to leave the standard of evaluation up to the respondents themselves. Whether, and to what extent, successful relationships play a role in evaluating their achievement of significant goals is left up to them. In this regard, the item on whether one could pursue personally meaningful goals to assess a fulfilled life permits persons to apply it to their preferences and life realities. Therefore, a person who has not been able or willing to start a family may still answer this item because it allows them to think in a broader context of personally meaningful goals. If a life goal has become unattainable (e.g., starting a family), the ability to adjust goals and pursue a new meaningful goal is associated with subjective well-being and a sense of purpose (Wrosch et al., 2013).

### Sources of fulfillment

#### Qualitative findings

Participants mentioned several aspects that could be considered sources of fulfillment. One main source is meaningful relationships and community in which people share and go through life together. The importance of this source has also been demonstrated by the finding that family and social relationships are predominant categories within contextual definitions of happiness and meaningfulness (Delle Fave et al., 2011) or that fulfilling personal relationships are an essential component of lay concepts of a good life (Twenge and King, 2005). Fulfillment derived from other social roles were partnership and parenting. A profession is another vital source of fulfillment, especially if the occupation is enjoyable, allows one to use one’s skills to contribute to others, and matches one’s calling. The theory suggests that viewing one’s occupation as a calling provides people with fulfillment (Wrzesniewski et al., 1997; Hall and Chandler, 2005). This assumption was recently confirmed by the finding that the perception of a calling was moderately to highly correlated with a fulfilled life (Baumann and Ruch, 2022). Other fulfilling areas included self-development, savoring, recreational activities, civic engagement, and spirituality. Overall, fulfillment stems from rewarding relationships, meaningful roles, and personally significant activities that allow for self-development and self-expression, but also from contributing to a cause beyond oneself.

#### Quantitative findings

Consistent with our qualitative findings, the quantitative results underscore the fact that multiple sources provide fulfillment. An interesting outcome is that participants rated nature as the most fulfilling category. There are several possible explanations for this result. Since people today are less likely to belong to a religious institution, the experience of connectedness to a larger whole could be found in nature. Indeed, connectedness with nature has been identified as a source of meaning and assigned to the subdimension of horizontal self-transcendence through a factor-analytic approach (Schnell, 2009). In addition, nature could be a place where people feel hopeful (Krafft and Walker, 2018) and experience a sense of beauty and excellence. The latter is a character strength attributed to the virtue of transcendence (Peterson and Seligman, 2004) and its application has been rated as fulfilling (Giuliani et al., 2020). Finally, a meta-analysis documented the positive relationship between nature connectedness and various indicators of happiness, such as positive affect, vitality, and life satisfaction (Capaldi et al., 2014). Our findings revealed that social and generative roles, including parenting, grandparenting, and volunteering, were also found to be very fulfilling. Volunteering and parenting were indeed positively correlated with a fulfilled life measured with the FLS (Baumann and Ruch, 2022). Underlying the three sources discussed is a perspective that transcends the self, implying that fulfillment comes from investing in the well-being of others. Learning and personal development were among the top five categories, suggesting the importance of expanding the self for experiencing fulfillment. Spirituality and religion was another category related to transcendence, but it was rated as less fulfilling than the other categories. However, for people who live their spirituality and religion in everyday life, this category had great relevance for their fulfillment, which was also confirmed in a study using the FLS (Baumann and Ruch, 2022).

The level of fulfillment provided by sources varied across age groups. In general, the two older age groups rated various sources as more fulfilling than the youngest age group. For instance, older adults valued the sources of nature and spirituality more highly. This is consistent with previous findings revealing that older individuals more often mentioned spirituality as a domain of happiness (Delle Fave et al., 2016) or that the sense of meaningfulness derived from vertical self-transcendence (religion, spirituality) increased with age (Schnell, 2009). Persons in the second half of life also rated learning and personal development as more fulfilling than their younger counterparts. Contrary to age stereotypes that are still prevalent, people enjoy learning even at advanced ages, as shown by recent meta-analyses that investigated age differences in character strengths (Heintz and Ruch, 2022). Having a life task and a profession were other sources that older participants valued more than younger participants. Even the oldest age group rated a profession as more fulfilling than the middle-aged persons. Although the need to matter and to have a meaningful engagement applies to individuals of different ages, it may be particularly essential for the well-being of older adults (Flett and Heisel, 2021). Overall, these findings suggest that as people age, they gain more self-knowledge about what is appropriate and meaningful to them, and thus learn to draw fulfillment from a variety of sources. It is also plausible that older adults are better able to pursue intrinsically motivated goals and have their psychological needs met (Bauer and McAdams, 2004; Mackenzie et al., 2018). Women considered friendships and social relationships as more fulfilling than did men. Research has demonstrated that women have more close relationships than men and are also personally more involved (Antonucci et al., 2004). In particular, assessing the sources of profession, life task, challenges, partnership and reaching a life goal as highly fulfilling went along with higher levels of a fulfilling life in the present and a fulfilled life in retrospect.

#### Integrating the findings

Most sources mentioned by participants were identical to our list of ratings. Although the ratings for nature and grandparenthood yielded high means in terms of the fulfillment they provide, they were rarely mentioned in the responses to the open-ended question. The reason for this could be that these aspects were not at the forefront when describing a fulfilled life. This underscores the added value of a mixed methods approach to gain a more complete understanding of fulfillment sources. The emergence of the category of savoring, on the other hand, was rather unexpected and the only one that was not among the listed sources of participants’ ratings. Savoring, which refers to the ability to note and appreciate positive experiences, can relate to the past, present, or future (Bryant et al., 2013) and is related to various positive psychosocial outcomes (Smith et al., 2014). In our study, all participants’ descriptions related to savoring in the present. Savoring might enable individuals to enhance the quality of their positive experiences (Bryant et al., 2013) and live their lives more consciously. Overall, insights on the nature of sources from both methodological approaches demonstrate the importance of a long-term perspective and having the willingness to commit and to invest over time (e.g., parenthood, achieving a life goal, career). Individuals draw fulfillment from sources that enable them to develop and flourish, but also from providing value to others’ lives.

#### Context of a fulfilled life

Various participants’ statements referred to prerequisites for a fulfilled life. On the one hand, requirements included psychological resources such as a healthy relationship with oneself, coping skills, and confidence. On the other hand, they comprised social resources, including a good start in life, a sense of being socially integrated, and having supportive relationships. A sense of belonging, for instance, helps people believe that their lives are meaningful (Lambert et al., 2013). Meaningful relationships support individuals in coping with adversity, as well as in their pursuit of opportunities for development and growth, which can have long-term effects on their flourishing (Feeney and Collins, 2015).

According to the participants, positive attitudes and personal qualities are essential for a life well lived. The personal qualities mentioned included positive character traits, personal agency, acceptance, and a positive attitude. These qualities can influence the extent to which one values life and what one makes of it. Participants mentioned curiosity above all, but also bravery and humility. These traits are also referred to as character strengths, which are positively valued personality traits that are thought to contribute to individual and collective fulfillment (Peterson and Seligman, 2004). In addition, it was suggested that strengths provide a basis for healthy processes and the attainment of fulfillment in both good and difficult times (Lopez and Snyder, 2003). Character strengths are known to be associated with life satisfaction (e.g., Baumann et al., 2020), and the excellent use of signature strengths has been found to be fulfilling (Giuliani et al., 2020). Moreover, character strengths can be cultivated to improve one’s well-being (e.g., Schutte and Malouff, 2019). Whether they also contribute to a fulfilled life in the long term needs to be determined through future research. Taking personal responsibility for creating a fulfilling life, accepting things one cannot change, and having a positive attitude were other personal characteristics mentioned.

A fulfilled life also seems to depend on resources and favorable conditions, some of which are beyond the individual’s control. In this context, participants referred to quality-of-life attributes, including health, financial well-being, individual freedom and opportunities, and a life in balance. There is ample evidence of the importance of factors such as opportunities to choose and income to people’s welfare and flourishing (e.g., Veenhoven, 2015) and, more recently, the relationship between a fulfilled life and contextual factors such as education, financial status, or self-rated health has been demonstrated (Baumann and Ruch, 2022). For example, lack of resources or health issues present barriers to living a calling (Duffy et al., 2017) and thus may hinder experiencing fulfillment in this life area. Therefore, a person’s particular circumstances may influence the extent to which they are able to realize their potential. It is also worth mentioning the aspect of luck, which was only addressed by men. The country in which one is born or whether one is spared from misfortune is due to luck and could frame the degree to which a fulfilling life is possible. Finally, it should be noted that a fulfilling life as an outcome variable can also be a resource, as it can promote positive attitudes or improve subjective health.

#### A fulfilled life with a view to the lifespan

Within the two age groups, younger participants more frequently mentioned a fully lived life, having had an abundant life, and having taken chances. These three topics relate to the FiL model’s criterion of wholeness/completeness and reflects that at a younger age, many dreams, goals, and life projects that fulfill a life are yet to be realized. With increasing life experience, individuals seem more aware that a person’s character, attitude, and initiative facilitate attaining a fulfilled life, as older people compared to younger more often referred to positive traits.

In their descriptions, participants referred to several aspects related to Erikson’s suggested developmental tasks that appear to facilitate a fulfilled life. Erikson (1985) proposed that healthy psychosocial development results from the successful resolution of eight specific psychosocial challenges across the lifespan, from each of which a psychological strength is gained. For instance, participants considered the personal development that has taken place throughout life and that has made them the person they are as fulfilling. They also mentioned a good childhood and confidence in life, which can be related to the developmental task of building trust, and which leads to the virtue of hope. Creating a stable sense of identity can provide the foundation for a fulfilled life. Erikson (1985) suggested that a clear sense of identity as a young adult serves as a basis for personal development at different stages of life, and the way individuals construct their identity influences adjustment outcomes (Beaumont, 2009). Berzonsky (1989) identified three identity styles: *informational* (e.g., seeking and evaluating self-relevant information), *normative* (e.g., conforming to expectations of close others), and *diffuse-avoidant* (e.g., circumventing identity conflicts and problems.) Failure to develop an integrated sense of one’s identity may hinder the active shaping of one’s life and the pursuit of goals consistent with oneself, which appear essential for a fulfilled life. The results of a path analysis indicate that only the informational identity style predicted self-actualization and self-transcendence in young adults, which in turn predicted meaning in life and subjective happiness (Beaumont, 2009). The informational style also appears to be associated with an orientation that exceeds self-interest; it is positively associated with the values of universalism and benevolence (Berzonsky et al., 2011). Finding a partner and maintaining a fulfilling partnership can be seen as parallels to the challenge of building lasting intimate bonds. The aspect of contributing to the lives of others or to a worthy cause is found in the task of becoming a generative person. Therefore, becoming a mature and adjusted personality involves the ability to be other-oriented and caring, which laypersons deemed an essential element of fulfillment (Ryff, 1989). Erikson (1985) considered these preceding stages as precursors to achieving ego integrity:

Only in him who in some way has taken care of things and people and has adapted himself to the triumphs and disappointments adherent to being, the originator of others or the generator of products and ideas—only in him may gradually ripen the fruit of these seven stages. (p. 268).

There is empirical evidence that successfully mastering earlier psychosocial challenges predicted ego integrity in older adults, with generativity proving to be the strongest predictor (Hannah et al., 1996). Psychosocial growth actually facilitated attaining a fulfilled life, as generativity and ego integrity were strongly associated with all dimensions of a fulfilled life (Baumann and Ruch, 2022). In addition, a small positive correlation was found between a fulfilled life and age, suggesting that older adults may look back on a journey in which they have grown, contributed to society, and accomplished personally meaningful projects.

#### Strengths, limitations, and future directions

To our knowledge, this is the first study to examine lay perspectives of a fulfilled life among adult participants across the lifespan. The fact that our sample included respondents from all life stages and had a gender balance can be considered a strength. However, the use of a convenience sample limits the generalizability of the findings. Since our sample consisted of German-speaking participants belonging to an individualistic culture, future research will have to establish whether conceptions of a fulfilled life differ across cultures, especially in comparison to collectivistic cultures. The numerous and valuable perspectives gained using both qualitative and quantitative data contribute to a deeper and more comprehensive understanding of a fulfilled life. By allowing participants to freely describe their conception of a fulfilled life, we obtained a rich, diverse, and in-depth collection of information, including conceptual components, correlates, and antecedents. Although we were able to gain a variety of perspectives and create a comprehensive framework of a fulfilled life, we cannot claim to have been exhaustive. Participants responded very carefully, personally, and with great depth. However, it is not possible to achieve the same depth with open-ended questions as with interviews, for example, which allow for further verification. Our cross-sectional design allows us to make statements about age-group differences, but not causality statements about age changes. Thus, age effects could be attributed to cohort membership, as typical mentalities of a particular cohort may shape the notions of a fulfilled life. It should be noted that significant age and sex differences are based on exploratory analyses without adjustments for multiple testing. Confirmatory studies will need to confirm these results. Finally, it should be noted that the first author, who also led the coding, was also involved in the development of the theoretical model of FiL (Baumann and Ruch, 2021). Although the principal investigator coded the data from the bottom up and high inter-rater reliability was achieved, it cannot be completely ruled out that prior knowledge may have influenced interpretation to some degree. A natural progression of this work would be to explore whether the lay-suggested antecedents (e.g., social resources, positive traits, or aspects regarding quality of life) are predictors of a fulfilled life.

#### Practical implications

Our overarching framework encompasses many routes by which a fulfilled life can be attained, whether by directly strengthening areas that are considered core elements or by fostering social and psychological resources, personal strengths, or positive attitudes. At the individual level, persons can reflect on and define what an ideally fulfilled life looks like for them and derive appropriate steps. As indicated in our conceptual model, it is essential to consider the core criteria. What is fulfilling depends on whether it contributes to perceiving wholeness, congruence, and significance. In addition, they can evaluate the sources that emerge from this study for their importance and cultivate those that provide the greatest fulfillment. In particular, evaluating the sources of profession and life task as fulfilling seems to have a longer-term impact on a fulfilling life in the present and a fulfilled life in retrospect. At any stage of life, it is worthwhile to pursue a career or activity that is a good fit or even perceived as a calling (Duffy et al., 2017; Baumann and Ruch, 2022). Leading a generative life seems to be a vital avenue to building a life of fulfillment and counteracts stagnation and the experience of emptiness (Erikson, 1982). Our findings also provide an important foundation for counseling on life and career planning. Knowledge of the elements, sources, or conditions of a fulfilled life can help counselors assist their clients in pursuing strategies that lead to a more fulfilling life. At the group and community level, efforts should be directed toward building strong marriages and healthy families, giving children a good start in life, and promoting positive education and organizations that create conditions for leading a fulfilling life.

## Conclusion

This study contributed to conceptual clarity by revealing what laypersons understand by a fulfilled life. Our qualitative results underscore that the construct is multidimensional and comprises cognitive and affective experiences. Statements that are considered cognitive appraisals indicate what matters to people in evaluating their lived lives. They understood a fulfilled life primarily as having lived life fully, achieved personally meaningful goals, developed themselves, and contributed to others and left a positive legacy. These findings confirm that a fulfilled life consists of many facets and, importantly, involves the notion that a life well lived contains a self-transcendent component. Lay perspectives regarding the core elements are largely consistent with the proposed theoretical conceptualization and the dimension of the FLS. Laypersons recognized that a fulfilled life also depends on personal resources, personal characteristics and quality-of-life aspects. The sources of fulfillment mentioned by participants and those given in the qualitative section largely overlap. It appears that fulfillment requires a long-term view, and that the quality and personal significance of the source are critical. As people age, they seem to succeed in drawing more fulfillment from the various sources. The understanding of what constitutes a fulfilled life is quite similar among younger and older adults and among men and women. The findings help lay the foundation for this emerging line of research and suggest many routes to create a more fulfilling life.

## Data availability statement

The raw data supporting the conclusions of this article will be made available by the authors, without undue reservation.

## Ethics statement

Ethical review and approval was not required for the study on human participants in accordance with the local legislation and institutional requirements. The patients/participants provided their written informed consent to participate in this study.

## Author contributions

DB and WR: conception and design of the work, interpretation of data analysis and final approval of the published version. DB: data collection, data analysis, and drafting of the manuscript. WR: critical revision of the manuscript. All authors contributed to the article and approved the submitted version.

## Conflict of interest

The authors declare that the research was conducted in the absence of any commercial or financial relationships that could be construed as a potential conflict of interest.

## Publisher’s note

All claims expressed in this article are solely those of the authors and do not necessarily represent those of their affiliated organizations, or those of the publisher, the editors and the reviewers. Any product that may be evaluated in this article, or claim that may be made by its manufacturer, is not guaranteed or endorsed by the publisher.

## Footnotes

^1^All quotes were translated from German into English.

^2^As data were collected without prespecified hypotheses, we did not include multiplicity correction. Significant results are to be considered as exploratory results.
