# Supplementary material for: What constitutes a fulfilled life? A mixed methods study on lay perspectives across the lifespan
Source: Front Psychol. 2022 Sep 30;13:982782. doi: 10.3389/fpsyg.2022.982782 (PMC9563392; doi:10.3389/fpsyg.2022.982782)
Supplement: Supplementary file 1 [file Table_1.docx]

Supplementary Material

# Table A1 | Content and frequencies of statements regarding a fulfilled life.

| LEVEL OF COMPONENT  Theme  Category  Subcategory | Content | Example quote (translated) | Number of statements (504) | % |
| --- | --- | --- | --- | --- |
| ELEMENTS |  |  |  |  |
| General description | A life that has enriched; can look different for everyone; a life considered fulfilled from an outside view can feel like a failure or vice versa; an old age constitutes fulfillment; reaching a high age allows one to judge about having had a fulfilled life; a fulfilled life clearly involves setbacks, as these bad feelings enable a comparison | “If I can say on my deathbed that I’ve had a fulfilling life, that would certainly be nice. That doesn't have to be a life of prosperity or publicity or..., I have to feel the life I lived was fulfilling. Was beautiful the life with all the ups and downs.” (Female, age 72) | 22 (12) | 4.37 |
|  |  |  |  |  |
| Positive evaluation | Positive things are in the foreground and prevail, one has done more right than wrong regarding one’s decisions and behavior, chosen path in life brought more joy than sorrow | “When I imagine that I am old and look back on my past life, I would like to come to the realization that despite all the mistakes I have made during my life, overall, I am satisfied with the way I have spent it.” (Male, age 24) | 10 |  |
| Cognitive appraisals |  |  | 199 | 39.48 |
| Fully lived life |  |  | 45 |  |
| Abundant life | Richness and wealth of experiences; has experienced a lot, had exciting encounters with people, could discover the world, and keeps many beautiful memories | “A fulfilled life is when you have seen and experienced many things.” (Female, age 21) | 20 |  |
| Realized dreams, desires | To have stuck to goals; visions, dreams were turned into reality | “Visions were turned into reality.” (Male, age 50) | 9 |  |
| Chances taken | Having dared, taken the opportunities | “The thought of having used one's chances … .” (Male, age 41) | 8 |  |
| Lived consciously | Having been present, enjoyed life, experienced life consciously | “A life that one has consciously led, in which one has consciously made decisions.” (Female, age 45) | 5 |  |
| Strived for | Having done what was possible, strived for happiness | “… and above all, you must always have striven for your own greatest happiness.” (Male, age 20) | 3 |  |
| Attained personally significant goals | Personal successes and achievements, goals attained | “A fulfilled life allows one to look back in old age on a large number of personal successes and achievements that were important to oneself. These are not necessarily financial successes, but rather inconspicuous things that perhaps only in retrospect turned out to be successes. Or paths despised by others, which one wanted to follow at all costs, which did not bring one to the planned goal, but perhaps to an unplanned, much greater goal.” (Female, age 52) | 43 (11) |  |
| Socially successful | Could find a partner, start a family, have a happy family, build up a solid and long-standing circle of friends and good relationships; see the positive development of the offspring | “So, for example, three healthy, intelligent and satisfied children, whom you have raised to become capable human beings and valuable members of society, is a symbol of a fulfilled life for me as a mother. If one has managed to maintain one’s partnership through ups and downs and to one’s own satisfaction, if one has struggled and suffered and yet is still together, then that is a symbol of a fulfilled life for me.” (Female, age 54) | 25 |  |
| Professionally successful | Satisfaction with what has been achieved professionally; having professionally attained what one wanted | “Satisfied with what has been achieved professionally.” (Male, age 53) | 7 |  |
| Developed self | How a person could learn, develop and grow; make progress regarding different aspects, learn from mistakes; experiment; develop and use strengths; redefine oneself | “I have become closer and closer to the person I want to be.” (Female, age 30) | 28 |  |
| Contribution, legacy | Having raised children; looking back on grandchildren and great-grandchildren; leaving an asset that generates profit across generations. Having passed on learnings from one’s life and what is essential for leading a good life; done something meaningful and valuable for society and the environment; helped people; given back; supported persons who were vital to themselves | “One has left traces. One has moved the world, even if only in small pieces.” (Male, age 54)  “… to be able to pass on one’s experiences, to be able to make a difference in society, or even just to be able to make a difference in one's environment.” (Male, age 41) | 24 |  |
| Having mastered life |  |  | 21 (1) |  |
| Mastered difficult life phases | Could master difficult life phases; cope with crisis; overcome low points in life | “A fulfilling life includes difficulties that had to be overcome. Without the endangerment of the circumstances recognized as valuable, I as a human being could not feel the emptiness as a counter design, from which a fulfilling life stands out.” (Male, age 34) | 10 |  |
| Made the best out of life events | Made the best out of negative life events, situations and setbacks and the knowledge of it | “His life was not always easy, he worked hard. A few years before retirement, he became a recipient of disability pension due to an accident. But he knew how to make the best of every situation.” (Female, age 72) | 6 |  |
| Recognized redemption | The adverse turned into good; a painful experience yielded a strong sense of justice; something that one would have chosen differently turned out well | “All adversity turns into good.” (Female, age 45) | 4 |  |
| Recognizing meaning, purpose | Feeling that one has fulfilled one’s purpose in life; had a life task; was able to recognize meaning in everything or in one’s action in retrospect | “So, the certainty of having led a meaningful life.” (Male, age 44) | 11 |  |
| Suited life | A sense that one was true to oneself; made good decisions, had taken the right path in life; one could pursue own ideas and interests and find opportunities to combine them with talents and apply them | “That you have the feeling to have gone this right way in life, even if others wanted to influence you differently at that time.” (Female, age 45) | 9 |  |
| Lived according to own values | Could be there for others; be an example to others; act morally right for oneself; live honestly | “One has acted morally right for oneself.” (Female, age 55) | 9 |  |
| Gained better self-knowledge | Having recognized oneself and one’s inclination, weaknesses, and strengths more clearly | “In my opinion, a very fulfilling life is one in which you have recognized early on who you are and what you bring to the table in terms of talents, strengths, resources.” (Female, age 62) | 5 |  |
| Meaningful use of talents, resources | Time and energy were not wasted but the feeling that they were properly managed | “The feeling that time has been properly managed.” (Female, 32) | 4 |  |
| Affective appraisals |  |  | 52 (2) | 10.32 |
| Being in harmony | Being at peace and in harmony with oneself, life circumstances, or the environment | “A fulfilled life is when you are reconciled with your life in old age.” (Female, age 84) | 20 |  |
| Gratitude | Looking back on one’s life with great gratitude; feeling deep gratitude; thankfulness for what is and for the things received | “A full life contains much gratitude and appreciation.” (Female, age 32) | 10 |  |
| Various positive emotions | Pride; carrying peace and serenity within; looking back with joy on what one has lived | “Inner peace” (Female, age 40) | 7 |  |
| Contentment | Great inner contentment; contentment with oneself, one’s life, what one has experienced; become a contented person | “The focus of the review is contentment.” (Female, age 35) | 7 |  |
| Widely free of regret | Looking back on without regret for not having done something; not regretting important decisions and experiences; not having to regret anything (in retrospect) | “For me, a fulfilled life is when I could die without regrets, with a great inner satisfaction.” (Female, age 30) | 6 |  |
| CORRELATES |  |  |  |  |
| Sources |  |  | 97 | 19.25 |
| Relationships, Community | Quality and depth of relationships; sharing time and life | “Being able to walk a little way with other people in good fellowship.” (Female, age 47) | 24 |  |
| Occupation | A fulfilling job, an occupation that one loves, provides joy, makes one happy, fits one’s calling, in which one can use one’s skills to add value to others | “I want professional fulfillment in a job where I add value to others through my skills.” (Female, age 32) | 18 |  |
| Recreational activities | Music; experiencing nature; sexual fulfillment; physical exercise; enjoying small things | “You can experience fulfillment in many things that you might not have expected.” (Female, age 52) | 12 |  |
| Learning, development | Getting to know new perspectives and people; being challenged in various tasks and obligations; learning new things | “Being challenged in various tasks and obligations.” (Female, age 66) | 9 |  |
| Partnership | Fulfilling partnership | “The person lives in a fulfilling partnership based on love and mutual respect.” (Female, age 52) | 9 |  |
| Savoring | Enjoying beautiful and magical moments | “… and if you can enjoy what is still possible.” (Female, age 84) | 9 |  |
| Spirituality | Fulfilling spirituality, living spirituality | “Lived spirituality, also in everyday life.” (Female, age 50) | 6 |  |
| Civic engagement | Work for a good cause, social engagement, charitable activities | “I also like to help other people who are worse off than me. All this is important for me to have a fulfilled life.” (Male, age 63) | 5 |  |
| Parenting | Fulfillment through parenting when raising children and watching them grow | “raising a child” (Female, age 49) | 5 |  |
| ANTECEDENTS |  |  |  |  |
| Quality of life |  |  | 48 (2) | 9.52 |
| Health | Being healthy | “Good health in all respects.” (Female, age 50) | 16 |  |
| Financial well-being | Having enough money; no existential hardships; financial security | “A fulfilled life is also associated with financial independence.” (Female, age 54) | 12 |  |
| Individual freedom, opportunities | Chance to decide independently; free choice of profession and where to live; opportunity to receive a good education | “I grew up in a time that offered me excellent opportunities, possibilities and conditions for a fulfilling life.” (Male, age 70) | 8 |  |
| Life in balance | Enough free time; balance between professional and personal life | “For me, a fulfilled life means being able to combine career and family without burning out.” (Female, age 71) | 6 |  |
| Luck, being spared | Living in peace, luck | “… and always a good portion of luck.” (Male, age 78) | 4 |  |
| Personal characteristics |  |  | 44 | 8.73 |
| Positive traits | Being grateful for one’s life and what one has; remaining humble and realistic; being brave; never giving up | “Sometimes it takes enormous ambition, sometimes overcoming, sometimes laissez-faire, sometimes discipline. Everything in moderation in the right place.” (Female, age 52) | 17 (10) |  |
| Curiosity | Always be curious, trying out new things | “always be curious” (Male, age 66) | 7 |  |
| Personal agency | Taking self-responsibility; developing creative initiative; being active and shaping one’s life; not blaming anyone for one’s happiness; the ability to say no | “You can't live a full life without doing it yourself.” (Female, age 75) | 13 |  |
| Acceptance | Accept things in life one would do differently, accepting what is | “Also being able to accept things in life that you would do differently today.” (Female, age 83) | 9 |  |
| Positive attitude | Seeing the positive; being able to put negatives into perspective | “We see everything with somewhat positive attitudes.” (Male, age 93) | 5 |  |
| Resources |  |  | 42 | 8.33 |
| Social |  |  | 29 |  |
| Social integration | Having loving people around; a stable family; good friendships; enjoying a trusting network of relationships in and outside the family; a sense of belonging | “… socially integrated are important prerequisites.” (Female, age 71) | 12 |  |
| Good childhood | Childhood that was carefree and sheltered, not overprotected; one has experienced love; felt taken care of; has experienced support | “Childhood in which the child is seen as his or her own personality well-cared for not over sheltered childhood.” (Female, age 60) | 10 |  |
| Social support | Having a family as a harbor from stormy times and who gives stability; friends one can rely on, who one can count on and who stand by one’s side; receiving support from different people | “… socially integrated are important prerequisites.”(Female, age 71) | 7 |  |
| Psychological |  |  | 13 |  |
| Healthy connection to oneself | Connectedness with oneself; self-forgiveness; self-acceptance | “When you don't let yourself be too unsettled by external factors in difficult times and have found a secure anchor within yourself.” (Female, age 42) | 7 |  |
| Can master difficulties | Ability to cope with difficulties and find solutions | “She copes well with the difficulties of life …” (Female, age 52) | 3 |  |
| Leading life with (self-) confidence | Confidence in life, going through life with confidence and self-confidence | “I go through life with confidence and self-assurance.” (Male, age 58) | 3 |  |

*Note*. Number in brackets in the column statements refers to the direct mention of the theme or category.
